# B-cell targeted therapies in autoimmune encephalitis: mechanisms, clinical applications, and therapeutic potential

**DOI:** 10.3389/fimmu.2024.1368275

**Published:** 2024-03-18

**Authors:** Haodong Shang, Xinru Shen, Xiaoxiao Yu, Jing Zhang, Yongliang Jia, Feng Gao

**Affiliations:** ^1^ Department of Neuroimmunology, Henan Institute of Medical and Pharmaceutical Sciences, Zhengzhou University, Zhengzhou, Henan, China; ^2^ BGI College, Zhengzhou University, Zhengzhou, Henan, China

**Keywords:** autoimmune encephalitis, B cell targeted therapies, monoclonal antibodies, chimeric antigen receptors, immunotherapy

## Abstract

Autoimmune encephalitis (AE) broadly refers to inflammation of the brain parenchyma mediated by autoimmune mechanisms. In most patients with AE, autoantibodies against neuronal cell surface antigens are produced by B-cells and induce neuronal dysfunction through various mechanisms, ultimately leading to disease progression. In recent years, B-cell targeted therapies, including monoclonal antibody (mAb) therapy and chimeric antigen receptor T-cell (CAR-T) therapy, have been widely used in autoimmune diseases. These therapies decrease autoantibody levels in patients and have shown favorable results. This review summarizes the mechanisms underlying these two B-cell targeted therapies and discusses their clinical applications and therapeutic potential in AE. Our research provides clinicians with more treatment options for AE patients whose conventional treatments are not effective.

## 1 Introduction

Autoimmune encephalitis (AE) is a group of diverse inflammatory autoimmune diseases that affect the brain parenchyma (cortex or deep gray and white matter) and may involve the meninges and spinal cord (1). AE accounts for 10%–20% of encephalitis cases, with an annual incidence of approximately 1 in 100,000 (2). Its clinical features include acute- or subacute-onset seizures, cognitive impairment, and psychiatric abnormalities.

B cells play a pivotal role in the pathogenesis of AE by the production of autoantibodies. Based on the location of the targeted antigen, AE-associated autoantibodies can be categorized as antibodies against neuronal cell surface and intracellular antigens. Among these, anti-neural surface protein antibodies have clear pathogenicity. However, the mechanism by which anti-intracellular antigen antibodies induce disease is unclear, although they may result from a non-dependent function of the antibodies. Patients positive for autoantibodies exhibit typical clinical features and can be diagnosed and clinically classified. For patients with anti-neurosurface protein antibodies, antibody-mediated encephalitis can lead to disease through different pathogenic mechanisms (3). Patients with clinical features of AE but no detectable autoantibodies are defined as having serum and cerebrospinal fluid negative AE (SCNAE), which can only be diagnosed based on clinical manifestations and auxiliary examinations (cerebrospinal fluid [CSF], magnetic resonance imaging, and electroencephalogram) (4).

AE can be categorized into several types, which display different pathophysiologies. Among these types, N-methyl-D-aspartate receptor (NMDAR) AE is the most common, accounting for approximately 54%–80% of AE cases, followed by leucine-rich glioma-inactivated protein 1 (LGI1) AE and gamma amino butyric acid type B receptor (GABA_B_R) AE (5). The characteristic clinical manifestations of NMDAR AE are consistent with features indicative of diffuse encephalitis, whereas glutamic acid decarboxylase (GAD) AE, LGI1 AE, and contactin-associated protein-2 (CASPR2) AE are consistent with limbic encephalitis.

The presence of antibodies in patients with AE reflects disruption of the CNS and/or peripheral tolerance mechanisms. In the bone marrow, impaired clearance of autoreactive B cells due to the disruption of negative selection barriers causes autoantibody-producing B cells to mature and subsequently differentiate into plasma cells (6).

The blood-brain barrier (BBB) makes it difficult for large molecular to enter the brain, but autoantibody can be detected in CSF of patients with AE.The influence of factors such as infection, brain injury, and emotional state may lead to increased permeability of the BBB, allowing autoantibodies to enter the brain (7–9). Compartmentalized enrichment of numerous CD20+ B cells and CD138+ plasma cells detected in brain tissue biopsies of patients with NMDAR AE indicate that autoantibodies in the CSF may be produced by activated immune cells entering the CNS (10–12).

The 2016 AE clinical criteria emphasize the importance of early immunotherapy once AE is highly suspected and infectious etiologies are excluded based on CSF results (4). In the acute phase, high-dose steroids are preferred in first-line immunotherapy, followed by the combination of steroids and intravenous immunoglobulins (IVIG) and/or plasma exchange. When first-line therapy fails, one converts to second-line immunotherapy. Rituximab (RTX) is considered the first-choice treatment at this stage, with cyclophosphamide subsequently considered (13). Cyclophosphamide is preferred over RTX when AE is associated with paraneoplastic syndromes because it can deplete T cells, cross the BBB, and may be part of the tumor treatment (14). In addition, patients with different antibody-positive AE may have different responsiveness to drugs, which may affect the choice of treatment regimen (15). Due to the clinical applicability and limitations of first- and second-line drugs, some patients with refractory disease cannot be treated promptly and effectively, which may lead to further disease progression and prolonged intensive care unit hospitalization.

Immunosuppressive therapy can reduce antibody levels in patients, but B cells still exist and produce antibodies. Since the 1990s, studies have demonstrated the effectiveness of therapies aimed at eliminating antibody-secreting cells (B cells or plasma cells) in treating autoimmune diseases. Currently, B-cell targeted therapies mainly involve two approaches: monoclonal antibody (mAb) therapy and chimeric antigen receptor T-cell (CAR-T) therapy. However, the use of these therapies in AE has not yet been summarized. In this review, we summarize the mechanisms of B-cell targeted therapies and their applications in AE and provide an outlook on their future development.

## 2 mAb therapy in AE

**Figure 1:**
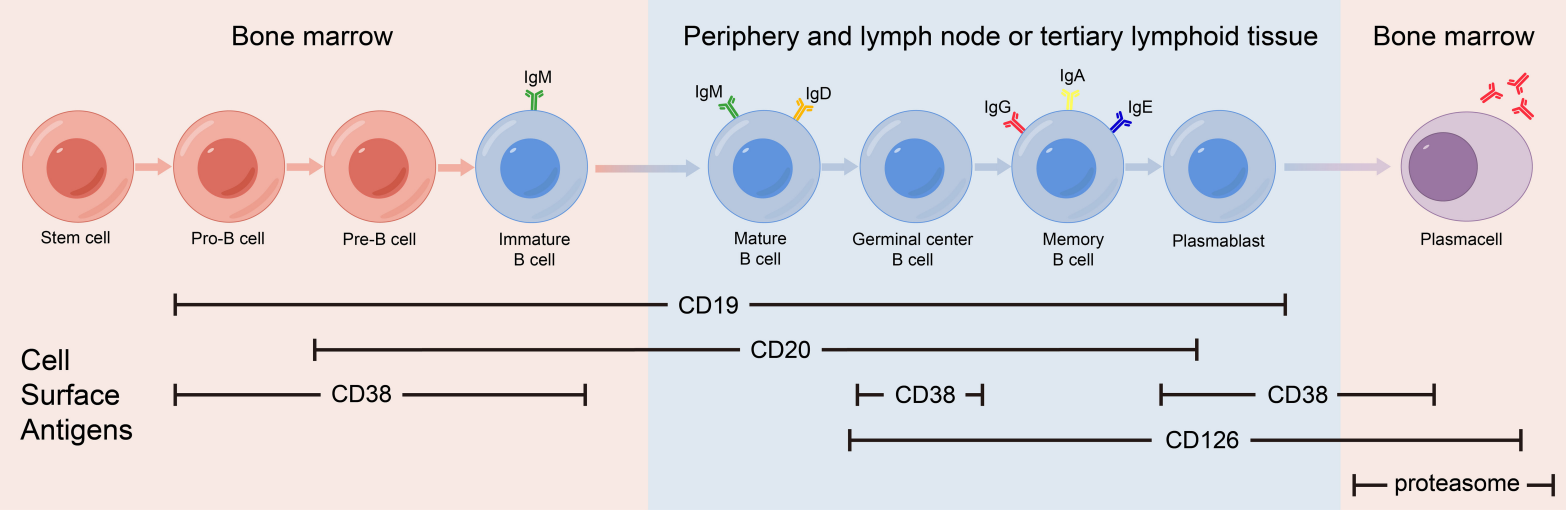
Expression of cell surface antigens during B-cell differentiation.

**Figure 2:**
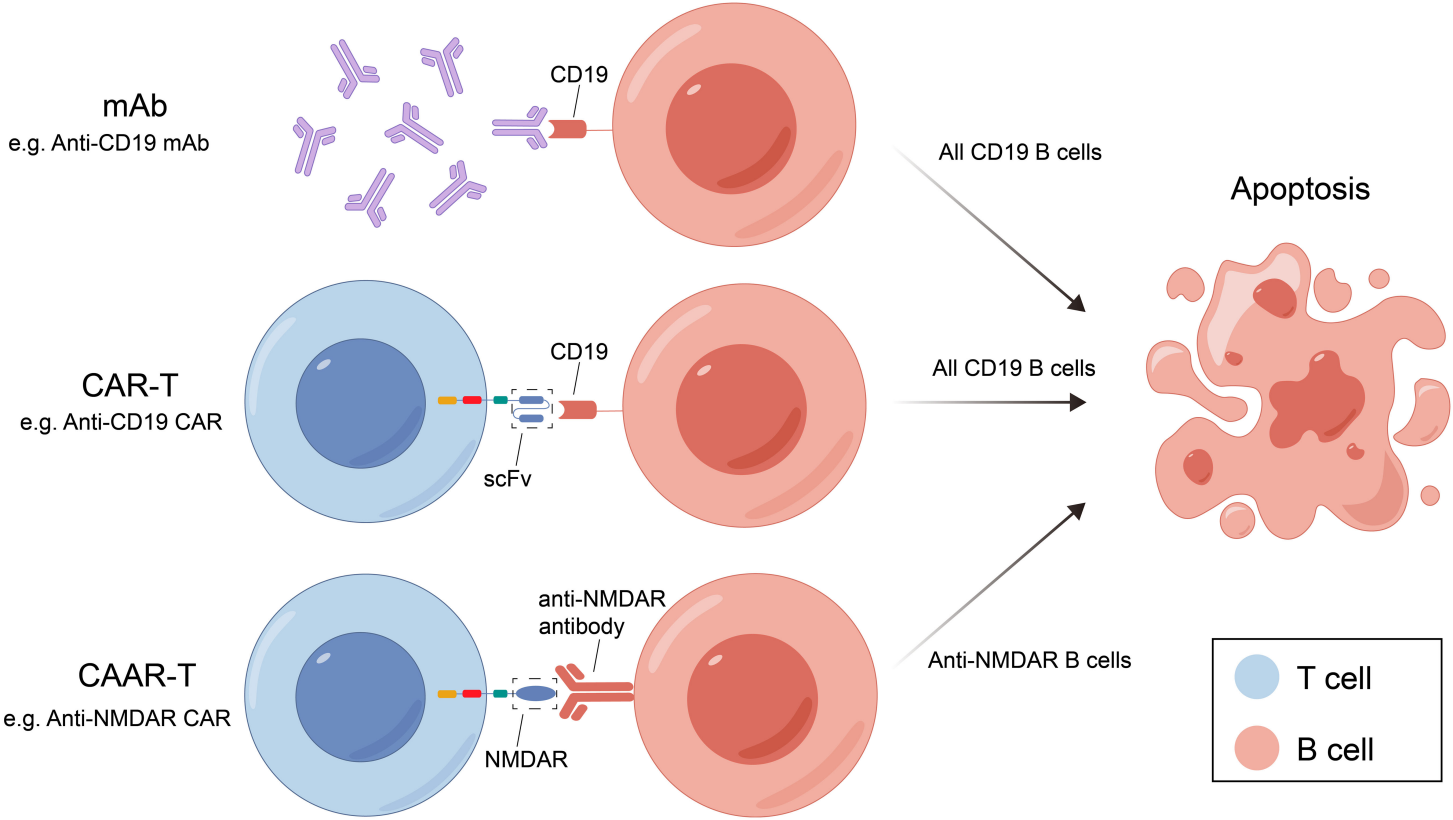
Three B-cell deletion therapies and their mechanisms. mAB, monoclonal antibody; CAR-T, chimeric antigen receptor T cell; RTX, rituximab; CAAR-T, chimeric autoantibody receptor T cell; NMDAR, N-methyl-D-aspartate receptor; scFv, single-chain variable fragment.

mAb therapy was initially employed for B-cell malignancies and has since been widely used for various autoimmune diseases. Many membrane molecules on the surface of B cells play important roles in B-cell recognition of antigens, activation, proliferation, and antibody production. Moreover, these roles constantly change during B cell development (**Figure 1**). mAbs can bind to these molecules, disrupt signaling pathways, and employ complement-dependent cytotoxicity (CDC) and antibody-dependent cellular cytotoxicity (ADCC) to reduce the number of antibody-producing cells and ultimately alleviate patient symptoms (**Figure 2**) (16). In recent years, mAb therapies targeting B cells have been widely used to treat AE (**Table 1**). Among these, RTX is the most extensively applied, serving as a second-line treatment for AE, while other mAbs are still in the early stages of exploration.

**Table 1:** B-cell targeted mAb therapies in use or under investigation for the treatment of AE.

Drug	Specific target	Evidence	Studied group; n	administration	Efficacy		Relapse, n (%)	Adverse, n (%)
					mRS score	CASE score		
Rituximab	CD20	Meta-analysis (17)	NMDAR AE:277	375 mg/m^2^ weekly for 4 weeks (most commonly used regimen)	mRS score ≤ 2; n (%) 206 (72) mean mRS score decreased by 2.67	NA	21 (14.2)	Infusion-related reactions: 29 (16); Pneumonia:11 (6); Severe sepsis:2 (1)
		Observational cohort (18)	NMDAR AE: 81 LGI1 AE: 26 CASPR2 AE: 11 GAD65 AE: 31	Infusion dose,g; Median (IQR) NMDAR:1.0(0.3) LGI1:1.0(0.3) CASPR2:1.0(0.02) GAD65:1.0 (0)	mRS score ≤2; n (%) NMDAR: 48 (94) LGI1: 20 (83) CASPR2: 4 (80) GAD65: 14 (52)	NA	NMDAR: 13 (19) LGI1: 5 (20) CASPR2: 1 (11) GAD65: 0	Infusion-related reactions: 2 Lymphopenia: 1 frequent infections: 1 unknown side effect: 1
Ocrelizumab	CD20	Terminated (NCT03835728) (19)	LGI1 AE: 1 (Patient 1) NMDAR AE: 2 (Patient 2-3)	Two 300-mg infusions 2 weeks apart, 600-mg infusion 24 weeks later; If clinical worsening endpoint is reached in the first 6 months, receive a single dose of 600 mg	Change of mRS: Patient 1: 3 to 3; Patient 2: 2 to 1; Patient 3: 4 to 1	NA	No	No
Ofatumumab	CD20	Case Series (20)	LGI1 AE: 2 (Patient 1-2) NMDAR AE: 1 (Patient 3)	Patient 1 and 2: 20 mg/week, 3 weeks Patient 3: 20 mg infusions 2 weeks apart	Change of mRS: Patient 1: 3 to 1; Patient 2: 2 to 1; Patient 3: 4 to 1	NA	No	short-term low-grade fever: 3 (100)
Inebilizumab	CD19	Ongoing (NCT04372615)	NMDAR AE	NA	NA	NA	NA	NA
Daratumumab	CD38	Case Report (21–23)	NMDAR AE: 2 (Patient 1-2) CASPR2 AE: 3 (3–5) SCNAE: 2 (6–7)	16 mg/kg weekly for the first 8 cycles, biweekly or monthly for the remaining cycles	Change of mRS Patient 1: 5 to 1 Patient 2: 5 to 3 Patient 3-5: 5 to 6 Patient 6: 5 to 5 Patient 7: 5 to 4	Change of CASE Patient 1: 27 to 1 Patient 6: 22 to 8 Patient 7: 21 to 3	No	Death after septic shock: 2 (67) Blood stream infections: 3 (100) urinary tract infections: 3 (100) Tracheobronchitis: 3 (100) Fever: 2 (67) Dyspnea: 1 (33) Tachycardia: 1 (33)
Bortezomib	Proteaso-me	Systematic review (24)	NMDAR AE: 29	Usually used 1.3 mg/m^2^ administered subcutaneously per cycle	mRS score ≤ 2; n (%) 16 (55)	NA	NA	Hematological side effects: 8 (28) infectious side effects: 3 (10) Gastrointestinal: 3 (10)
		Ongoing (NCT03993262)	No antibody specified AE	NA	NA	NA	NA	NA
Tocilizumab	IL-6R	Case report (25)	CASPR2 AE: 1	8 mg/kg every 4 weeks	Full remission	NA	No	No
		Retrospective cohort (26)	No antibody specified AE: Tocilizumab group:30 Additional rituximab group:31	Tocilizumab group: 8mg/kg monthly for >= 2 cycles Additional rituximab group: Tocilizumab 8mg/kg + rituximab 375 mg/m^2^ monthly	mRS score ≤ 2; n (%) Tocilizumab group: 18 (60) Additional rituximab group: 7 (23)	NA	NA	Tocilizumab group: decrement of absolute neutrophil count: 3 (10)
		Prospective cohort (27)	NMDAR AE: 52	Teratoma removal, Steroid, IVIG, Rituximab, and Tocilizumab (Tocilizumab: 8 mg/kg monthly)	In the subgroup analysis with baseline mRS score of 5 (n=15), 1-year Δ mRS scores: 4	In the subgroup analysis with baseline of 5 (n=15), 1-year Δ CASE scores: 21	NA	Pneumonia: 6 Neutropenia: 11 Lymphopenia: 5 Urinary tract infection: 7 Any serious adverse event: 1
Satralizumab	IL-6R	Ongoing (NCT05503264)	NMDAR/LGI1 AE	NA	NA	NA	NA	NA

AE, Autoimmune encephalitis; CAAR-T, chimeric autoantibody receptor T cell; IL, interleukin; NMDAR, N-methyl-D-aspartate receptor; LGI1, leucine-rich glioma-inactivated protein 1; CASPR2, contactin-associated protein-like 2; GAD, glutamic acid decarboxylase; SCNAE, serum and cerebrospinal fluid negative AE; NA, not available; CASE, Clinical Assessment Scale for Autoimmune Encephalitis; mRS, Modified Rankin Scale.

### 2.1 mAbs targeting CD20

CD20 is a transmembrane protein that may function by indirectly regulating calcium dependent on B cell receptors, thereby regulating B cell proliferation and differentiation (28). CD20 is expressed in pre-B cells in the bone marrow, naïve B cells in lymphoid tissues or germinal centers, and memory B cells, but not in hematopoietic stem cells, pro-B cells, most plasmablasts and antibody-producing plasma cells.

#### 2.1.1 RTX

RTX is a mouse-human chimeric mAb of the IgG1 class that binds to amino acid residues in the extracellular loop of CD20. RTX exerts its effects via CDC and ADCC, with CDC playing a predominant role. The Fab variable regions of RTX are all mouse-derived structures, so treatment with RTX may induce an immunogenic response, which may reduce therapeutic efficacy and/or lead to adverse events (29). RTX was the first anti-CD20 monoclonal antibody approved for use against human diseases and is now widely used as a second-line treatment for AE.

A meta-analysis conducted in 2020 examined the efficacy and safety of RTX in AE (17). 277 patients from 14 studies included in the meta-analysis revealed favorable outcomes in 72.2% of patients after RTX treatment. Meanwhile, there were no significant differences in outcomes within different patient subgroups (e.g., adults and children, standard and low dose RTX, Asian and non-Asian, NMDAR and mixed antibodies AE). Meta-analysis of 7 studies reporting mRS at the last follow-up showed that the initiation of RTX treatment significantly reduced the average Modified Rankin Scale (mRS) score by 2.7 points. A significant correlation was found between disease duration at RTX initiation and reduction in mRS score (p=0.0071). Therefore, timely RTX administration is suggested when first-line treatment is ineffective. Meta-analysis of 7 studies reporting relapse showed a relapse rate of only 14.2%. In a subcohort of 184 patients from 11 studies reporting the adverse reactions associated with RTX, infusion-related reactions were reported in 29 patients (15.7%), pneumonia was reported in eleven patients (6.0%) and severe sepsis in two (1.1%).

A multicenter clinical study in 2021 suggested varied responses to RTX treatment among patients with AE and different antibody types (18). RTX showed significant efficacy in NMDAR, LGI1, and CASPR2 AE, while GAD65 AE exhibited less favorable treatment outcomes. The disease duration before using RTX affected the reduction of the mRS score, emphasizing the potential benefit of early RTX treatment in controlling disease progression.

#### 2.1.2 Ocrelizumab

Ocrelizumab is a humanized anti-CD20 mAb of the IgG1 class. It targets a different but overlapping epitope with RTX. Except for retaining part of the mouse structure of the complementarity determining regions on the Fab, the other parts of ocrelizumab are all human components. Therefore, the immunogenicity induced by Ocrelizumab is lower than RTX. Compared with RTX, ocrelizumab exhibits lower CDC activity but higher ADCC activity (28).

A study on the efficacy of ocrelizumab in AE (NCT03835728) was terminated because of the inability to reach target enrollment. In two reported cases of patients treated with ocrelizumab, both demonstrated an improvement in clinical symptoms. In the placebo group, one patient experienced clinical deterioration at 12 weeks, subsequently received ocrelizumab and showed improvement. No serious adverse reactions related to ocrelizumab were noted in any of the three patients (19).

#### 2.1.3 Ofatumumab

Ofatumumab(OFA) is a fully human mAb of the IgG1 class. Its immunogenicity is the lowest among all anti-CD20 mAb. Therefore, it has a favorable safety profile. OFA targets a different CD20 epitope from that of RTX, allowing it to dissolve RTX-resistant cells expressing CD20 (29, 30). *In vitro* studies in RTX-sensitive tumor cell lines have shown that OFA exhibits twice the ADCC activity and ten times the CDC activity compared to that of RTX (31). Furthermore, OFA has a stronger binding affinity and a slower dissociation rate from CD20 (29). Compared with RTX, OFA lasts longer in patients and is administered less frequently.

OFA has been approved by the US Food and Drug Administration (FDA) for the treatment of multiple sclerosis (MS); however, the use of OFA in AE has only been described in a 2023 study (20). Three patients with AE who were unresponsive to or ineligible for methylprednisolone and IVIG treatment were administered OFA injections. The patients showed an immediate reduction in CD20+ B cells and improvement in clinical symptoms. In patients with MS treated with OFA, the most common adverse reactions were injection-related reactions, including mild or moderate fever, headache, myalgia, chills, and flushing. In contrast, only short-term hypothermic injection reactions were observed, demonstrating the safety of OFA in the clinical treatment of AE.

### 2.2 mAbs targeting CD19

As the main signaling component of multi-molecular complexes on the surface of B cells, CD19 can form complexes with membrane proteins such as CD21 (complement receptor) and CD81. This complex lowers the threshold for antigen-triggered B-cell activation by modulating the B cell receptor (BCR) signaling pathway (32). CD19 is expressed from progenitor B cells to plasma cells, and its expression gradually decreases during differentiation. CD19 expression spans the entire differentiation stage of B cells and therefore can serve as a specific marker on the B cell surface.

While anti-CD20 mAbs can effectively deplete mature naïve and memory B cells, B cells in the early developmental stages and most plasma cells that do not express CD20 are largely resistant to CD20 depletion (33). The anti-CD19 mAbs have a wider range of action and can eliminate B cells more completely.

#### 2.2.1 Inebilizumab

Inebilizumab is a humanized anti-CD20 mAb. In addition to the coding sequence of F(ab)_2_ was humanized to reduce immunogenicity, the affinity of the F(ab)_2_ moiety for CD19 was optimized, and its crystallizable fragment (Fc) was converted into human IgG1. Inebilizumab depletes CD19+ B cells primarily through ADCC (34). It is approved in 2020 by the FDA for the treatment of neuromyelitis optica spectrum disorder.

CD19+ is significantly expanded in B cells in patients with NMDAR AE and is markedly higher than that in patients with non-inflammatory neurological disorders, supporting the use of anti-CD19 mAb. However, the current application of inebilizumab in AE is limited to a phase IIb, double-blind, randomized controlled trial (NCT04372615) that is currently recruiting. This trial aims to evaluate the efficacy and safety of inebilizumab for the treatment of NMDAR AE.

### 2.3 mAbs targeting CD38

CD 38 is a multifunctional receptor and an extracellular enzyme found on the surface of B cell. It is expressed in the early B-cell development when stimulated by cytokines, endotoxins, and interferons. The expression levels of CD38 continue to change as B cells differentiate, eventually peaking in plasma cells. CD38 on the surface of B cells can affect B cell activation, induce apoptosis of immature B cells and promote B cell proliferation (35). CD38 is not a surface molecule unique to B cells, it is also expressed on monocytes, NK cells, and T cells.

#### 2.3.1 Daratumumab

Daratumumab is a humanized IgG1 mAb that primarily targets CD38 surface proteins in plasma cells. It can induce B-cell-associated tumor cell death through various mechanisms and is approved by the FDA for he treatment of multiple myeloma (36).

Seven patients with AE receiving daratumumab have been reported (NMDAR AE, n=2; CASPR2 AE, n=3; SCNAE, n=2). All patients showed varying degrees of clinical improvement (reduction in mRS or Clinical Assessment Scale in Autoimmune Encephalitis scores [CASE]) and a reduction in autoantibody titers and total IgG levels in the serum and CSF.

The adverse reactions resulting from daratumumab treatment included urinary tract infections, tracheobronchitis, bloodstream infections, fever, shortness of breath, and tachycardia. Three patients with CASPR2 AE died, two of whom died after developing gram-negative shock, which was considered a serious adverse reaction associated with the treatment regimen. Therefore, the impact of the drug’s side effects should not be ignored when using daratumumab to treat patients with AE (21–23).

### 2.4 mAbs targeting the proteasome

Proteasomes are widely found in the nuclei and cytoplasm of eukaryotic cells, with the most common being the 26S proteasome, which consists of a 20S core proteasome and two 19S regulatory proteasomes. The ubiquitin-proteasome pathway degrades intracellular proteins, in which ubiquitin marks the proteins that are slated for degradation and recognition by the proteasome. The nuclear factor kappa B (NF-κB) pathway is crucial for regulating both innate and adaptive immunity, wherein ubiquitin marks the inhibitory factor of NF-κB (IκB), leading to its degradation in the proteasome and subsequently activating NF-κB to induce cell activation and proliferation.

#### 2.4.1 Bortezomib

Bortezomib is a proteasome inhibitor that primarily targets the 20S core proteasome. It inhibits the ubiquitin-proteasome pathway by inhibiting proteasome activity, IκB cannot be degraded, thereby blocking NF-κB activity and inducing apoptosis, and is able to significantly deplete short- and long-lived plasma cells in the peripheral blood and bone marrow (37). RTX only depletes pre-B and mature B cells. However, long-lived plasma cells may continue to produce antibodies, potentially contributing to the lack of improvement observed in some patients following RTX treatment. Therefore, combined therapy with RTX and bortezomib, which can deplete both B and plasma cells, may be an option for the treatment of severe and refractory AE (38).

A 2021 systematic review summarized 29 patients with NMDAR AE treated with bortezomib with a mean age of 26 years, including six pediatric patients (<17 years old) and two elderly patients (≥50 years old). After demonstrating poor responses to various conventional first- and second-line treatments, these patients underwent 1–6 cycles of bortezomib treatment based on their condition and efficacy. Most patients showed decreased antibody titers in the serum and CSF after bortezomib treatment. The reduction in antibody titers was more significant in patients with favorable outcomes than in patients with unfavorable outcomes. Eleven patients experienced side effects after bortezomib treatment, among which hematological side effects were the most common, followed by infections and gastrointestinal disorders (24).

A phase II, multicenter randomized, double-blind, placebo-controlled trial (NCT03993262) is currently being conducted to evaluate the efficacy and safety of bortezomib in patients with severe AE (antibody type is not limited).

### 2.5 mAbs targeting IL-6R

Interleukin-6 (IL-6) is a typical multifunctional cytokine that plays a central role in acute inflammatory responses. IL-6 can be secreted by a variety of cells and its receptor system includes two components: the IL-6 receptor (IL-6R, also known as CD126) and Glycoprotein 130 (gp130). IL-6R can be expressed on activated B cells and plasma cells. IL-6 can be used as B cell stimulating factor to activates downstream signaling pathways, promote the differentiation of effector B cells into antibody-producing cells (39).

#### 2.5.1 Tocilizumab

Tocilizumab is a humanized anti-IL-6R mAb of the IgG1 class. It can inhibit signal transduction by blocking the binding of IL-6 to IL-6R. Although it cannot directly delete B cells, it can indirectly reduce the number of antibody-producing cells through functional regulation, and ultimately reduce antibody levels (40).

Tocilizumab was first administered to a patient with CASPR2 AE, who responded well to treatment (25). The subsequent study by Lee et al. provided a basis for the application of tocilizumab in AE treatment. This 3-year retrospective institutional cohort study included 91 patients with AE who demonstrated no significant clinical response to first-line therapy and 1 month of rituximab treatment. The patients were divided into three groups: tocilizumab treatment (n = 30), additional rituximab treatment (n = 31), and observation (no further treatment, n = 30). Compared with the other two groups, tocilizumab showed better mRS improvement (mRS score reduction ≥2 points), and a more significant favorable clinical response at the final follow-up (mRS score improvement ≥2 points or mRS score ≤2 points). Moreover, among the 30 patients who received tocilizumab, none showed infections or infusion-related adverse reactions, and only three patients showed a decrease in absolute neutrophil counts (26).

Lee et al. also analysed the efficacy of combined treatment of teratoma removal, steroid, IVIG, rituximab and tocilizumab (T-SIRT) (27). In the subgroup analysis for the patients with baseline mRS score of 5, the combination immunotherapy that included tocilizumab significantly reduced CASE and mRS scores compared with those that did not when the treatment regimen is completed within one month. Decreased lymphocyte count also were observed in this study.Therefore, close follow-up of the complete blood count is required during tocilizumab treatment.

#### 2.5.2 Satralizumab

Satalizumab is a humanized IgG2 mAb. Satalizumab has the same mechanism of action as that of tocilizumab, which acts by blocking the IL-6 signaling pathway. However, it employs a novel circulating antibody technology that allows satralizumab to dissociate from the IL-6 receptor in a pH-dependent manner. IL-6R bound to the drug is degraded in lysosomes, and the drug is re-released into the plasma to combine with other IL-6R molecules, increasing the duration of action of satralizumab to four times that of tocilizumab (41).

A phase III randomized double-blind placebo-controlled multicenter basket study is currently underway (NCT05503264) to evaluate the efficacy, safety, pharmacokinetics, and pharmacodynamics of satralizumab in NMDAR and LGI1 AE.

## 3 CAR-T treatment of AE

CAR-T cell therapy was originally used as a tumor treatment method. Due to its good therapeutic effect on tumors, it has also been studied in autoimmune diseases. Genetic modification technology is used to produce CAR-T cell by transfering genetic material encoding sequences of specific antigen recognition domain (e.g., single chain variable fragment) and intracellular activation signal. T cells expressing chimeric receptors are activated by directly combining with specific antigens on the surface of target cells and killing the target cells directly by releasing perforin, granzyme B, etc. T cells also recruit endogenous immune cells to kill pathogenic cells by releasing cytokines for disease treatment and can also form immune memory T cells.

Chimeric autoantibody receptor T cells (CAAR-T) therapy have been investigated for the treatment of B-cell-related autoimmune diseases. The mechanism of CAAR-T is similar to CAR-T, but its specific antigen recognition domain is replaced by specific antigen so that CAAR-T cells can specifically recognize and kill antigen-specific B cells and reduce autoantibody production without harming other B cells, thus reducing the cytotoxicity of CAR-T therapy (**Figure 2**).

Reincke et al. developed NMDAR-specific chimeric autoantibody receptor T cells (NMDAR CAAR-T), which contain NMDAR autoantigen fragments fused to the CD8 hinge and a 4-1BB/CD3ζ intracellular signaling domain. *In vitro* experiments, K562 and Nalm6 cell lines co-expressing human mAb and luciferase (ffluc) were used as target cells to activate and expand NMDAR CAAR-T cells. Activated NMDAR CAAR-T cells can secrete interferon-gamma and granzyme B to specifically lyse target cells, and ffluc was used to verify the killing effect of NMDAR CAAR-T cells. *In vivo* experiments, NMDAR antibodies and Nalm6 cells were co-injected into immunodeficient mouse lacking natural killer cells and lymphocytes, followed by injection of NMDAR CAAR-T cells for treatment. Reduction of Nalm6 cells and elimination of antibodies was observed, with no evidence of off-target toxicity or other adverse effects. However, Nalm6 cells belong to the lymphoblastoid cell line, representing precursors of B cells, while the goal of CAAR-T is the specific elimination of antibody-secreting B cells. Meanwhile, this study did not establish an AE animal model to verify the efficacy of CAAR-T under real pathological conditions. The vivo experiment only monitored the efficacy and off-target effects of CAAR-T within 20 days, and did not study the duration of its effect (42).

The results of preclinical models investigating the efficacy and safety of NMDAR-CAART supported its potential as a precise cellular immunotherapy for inducing complete and durable remission in NMDAR AE and provided a basis for phase I/II trials of CAAR-T cells in NMDAR and other AE.

## 4 Discussion

### 4.1 Current challenges in AE treatment

In this article, we described the mechanisms of mAb and CAR-T therapies and discussed the existing evidence for the treatment of AE. Both approaches have advantages and disadvantages. In terms of production, mAbs are universally available, whereas CAR-T cells must be produced individually based on each patient’s T cells, resulting in higher costs. In clinical use, mAbs need to be administrations multiple times to achieve the desired and stable results owing to their short half-life, whereas CAR-T cells can self-amplify in the body and last longer. While CAR-T cells demonstrate superior B cell depletion compared to that by mAbs, fludarabine and cyclophosphamide are required for lymphocyte clearance before treatment. The stability and persistence of CAR-T cells in the body vary among individuals, leading to inconsistent clinical outcomes. The BBB prevents easy entry of mAbs into the CNS, whereas CAR-T cells exhibit better tissue penetration. After peripheral injection, CAR-T cells enter the CNS and exert their effects (43). Regarding safety, the common adverse events associated with mAb include immune reactions, infections, hematologic events, and infusion-related reactions (44). For CAR-T cell therapy, cytokine release syndrome is the most common but can be addressed using drugs that inhibit cytokine secretion or block cytokine signal transduction. The risk of T-cell exhaustion increases owing to sustained antigen stimulation, and severe risks of T-cell malignancies have been reported in patients treated with CAR-T cells targeting B-cell maturation antigen (BCMA) or CD19. Caleb et al. reported reactivation of human herpes virus 6 in CAR-T cell-treated patients based on genomic analysis (45). Both mAb and CAR-T therapies targeting B-cell surface molecules can lead to broad immunosuppression, whereas CAAR-T therapy targeting specific antibodies has a more precise action, avoiding immunosuppression-induced side effects.

Many questions remain regarding the current exploration of B-cell targeted therapies in AE. Currently, research on mAb and CAAR-T therapies mainly focuses on NMDAR AE, but different antibodies of AE exhibit distinct clinical characteristics. However, studies on other types of AE are limited. Additionally, AE is a rare disease, which limits the number of included patients. The feasibility of mAb treatment for AE has only been explored in a small number of patients, and CAAR-T therapy remains in the preclinical stage. Large-scale randomized controlled trials are required to validate these treatments.

### 4.2 Current status and prospects of B-cell targeted therapy

The efficacy of various mAb in patients with different types of AE is summarized in **Table 1**. In NMDAR AE cases, the effectiveness of RTX, Bortezomib and Tocilizumab have been demonstrated their effectiveness in large clinical studies, whereas Ocrelizumab, OFA and Daratumumab has only been studied in individual cases. For LGI1 AE, RTX and OFA are effective, but the effectiveness of ocrelizumab is not significant. Treatment of CASPR2 AE with RTX and Tocilizumab is effective, but the use of Daratumumab can cause serious adverse reactions and even death. For GAD65 patients, only the effectiveness of RTX has been studied, but it is not significant. Additionally, Daratumumab and Tocilizumab have shown significant therapeutic effects on SCNAE and no-antibody-specified AE, respectively.

In addition to the mAbs mentioned in the article, mAbs currently used in the treatment of cancer or other autoimmune diseases also show significant potential for the treatment of AE. For example, epratuzumab, which targets CD22 expressed on developing B cells, except plasma cells and plasmablasts, showed no difference in effectiveness from the standard treatment in phase III clinical trials in patients with systemic lupus erythematosus (46–48). B-cell activating factor (BAFF) can induce autoimmune diseases when overexpressed (49). mAbs targeting BAFF, such as belimumab and tabalumab, can prevent the binding of BAFF to its receptor system on the B cell surface and the differentiation of B cells (50).

Furthermore, interactions between T and B cells in AE cannot be ignored. Blocking the interactions may also be a treatment strategy for AE. Li et al. observed a significant decrease in the expression of regulatory B cells (Bregs) and regulatory T cells (Tregs) and an increase in T follicular helper cells (Tfh) in patients with NMDAR AE, indicating that Bregs may also be involved in AE pathogenesis (51). Bregs do not have a distinctive transcription factor and are characterized by the production of the inhibitory cytokine IL-10. IL-10 inhibits the proliferation and differentiation of effector T cells, including Tfh cells, and increases the number of Tregs. Tfh play a crucial role in germinal center formation, B-cell differentiation into plasma and memory cells, and antibody production. Therefore, increasing IL-10 expression may be a potential therapeutic approach for AE.

Another T cell-B cell interaction is that of CD40 and its ligand (CD40L). CD40 is expressed on B cells and other antigen-presenting cells, whereas CD40L is expressed on activated T cells (52). In the acute phase of NMDAR AE, serum CD40 levels decrease, whereas CD40L levels increase and the CD40L/CD40 ratio decreases. In the remission phase, the CD40 and CD40L levels and the CD40L/CD40 ratio decrease. Thus, the CD40/CD40L signaling pathway may be involved in the pathogenesis of NMDAR AE (53). Blocking the CD40/CD40L pathway may reduce the antigen-presenting ability of cells and inhibit B cell proliferation, differentiation, and antibody class switching, thereby reducing antibody production.

No studies have yet reported on the effectiveness and safety of CAR-T cell therapy targeting universal targets in AE. Considering the favorable performance of mAbs targeting CD19/20 in AE, similar effects are expected for CAR-T therapy. The development and preclinical work on NMDAR-CAAR T cells provides a solid foundation for phase I/II trials of CAAR T cells in NMDAR AE and offers valuable insights for designing CAAR T cells for other AE.

CAR-Tregs recognize specific antigens, which leads to their activation and proliferation. CAR-tregs can directly deactivate antigen-presenting cells to prevent their presentation to T cells. CAR-Tregs can also inhibit the proliferation and differentiation of effector B cells and T cells by producing inhibitory cytokines (transforming growth factor-beta, IL-10, and IL-35). Finally, CAR-Tregs suppress rejection reactions by releasing cytotoxic granules and perforin proteins to kill effector B and T cells, thus indicating the feasibility of applying CAR-treg cell therapy for AE.

Further design and optimization of CAR structures are necessary to enhance the specificity, affinity, and persistence of CAR-T cells. Simultaneously, improving the immunotolerance of CAR-T cells to reduce the adverse reactions associated with CAR-T therapy should also be considered. Although CAR-T therapy has progressed to the fifth generation, second-generation CAR-T cells remain the primary therapy owing to their effectiveness and safety concerns. Given the high cost of CAR-T cell production, CAR-T cells should be developed that can be rapidly produced to reduce the treatment time and costs, thereby alleviating patient burden.

In conclusion, B-cell targeted therapy has broad application prospects in the treatment of AE. Researchers should delve deeper into understanding disease mechanisms, developing safer and more effective B-cell targeted therapies, and providing more accurate, timely, and personalized treatment options for AE.

## Author contributions

HS: Conceptualization, Software, Writing – original draft, Writing – review & editing. XS: Data curation, Writing – review & editing. XY: Writing – original draft, Writing – review & editing. JZ: Supervision, Writing – review & editing. YJ: Methodology, Software, Writing – review & editing. FG: Funding acquisition, Writing – review & editing.
